# JMJD6 Regulates Splicing of Its Own Gene Resulting in Alternatively Spliced Isoforms with Different Nuclear Targets

**DOI:** 10.3390/ijms21186618

**Published:** 2020-09-10

**Authors:** Nikoleta Raguz, Astrid Heim, Eden Engal, Juste Wesche, Juliane Merl-Pham, Stefanie M. Hauck, Steffen Erkelenz, Heiner Schaal, Olivier Bensaude, Alexander Wolf, Maayan Salton, Angelika Böttger

**Affiliations:** 1 Department of Biology II, Ludwig Maximilians University, Munich, Großhaderner Strasse 2, 82152 Planegg-Martinsried, Germany; astridheim6868@aol.com; 2 Institute of Molecular Toxicology and Pharmacology, Helmholtz Zentrum München—German Research Center for Environmental Health, Ingolstädter Landstrasse 1, 85764 Neuherberg, Germany; juste.wesche@gmail.com (J.W.); Wolf.alex@web.de (A.W.); 3 Department of Biochemistry and Molecular Biology, Institute for Medical Research Israel Canada, Faculty of Medicine, Hebrew University of Jerusalem, Jerusalem 91120, Israel; eden.engal@mail.huji.ac.il (E.E.); maayan.salton@mail.huji.ac.il (M.S.); 4 Research Unit Protein Science, Helmholtz Zentrum München—German Research Center for Environmental Health, Ingolstädter Landstrasse 1, 85764 Neuherberg, Germany; juliane.merl@helmholtz-muenchen.de (J.M.-P.); hauck@helmholtz-muenchen.de (S.M.H.); 5 Institute for Genetics and Cologne Excellence Cluster on Cellular Stress Responses in Aging-Associated Diseases (CECAD), University of Cologne, 50931 Cologne, Germany; serkelen@uni-koeln.de; 6 Institute of Virology, Medical Faculty, Heinrich Heine University Duesseldorf, 40225 Duesseldorf, Germany; schaal@uni-duesseldorf.de; 7 Institute de Biologie de l’Ecole Normale Supériuere (IBENS), Ecole Normale Supérieure, CNRS, INSERM, PSL Research University, 5005 Paris, France; bensaude@bio.ens.psl.eu

**Keywords:** splicing, polyS domain, hydroxylation

## Abstract

Jumonji-domain-containing protein 6 (JMJD6) is a Fe(II) and 2-oxogluterate (2OG) dependent oxygenase involved in gene regulation through post-translationally modifying nuclear proteins. It is highly expressed in many cancer types and linked to tumor progression and metastasis. Four alternatively-spliced *jmjd6* transcripts were annotated. Here, we focus on the two most abundantly expressed ones, which we call *jmjd6-2* and *jmjd6-Ex5*. *TCGA SpliceSeq* data revealed a significant decrease of *jmjd6-Ex5* transcripts in patients and postmortem tissue of several tumors. The two protein isoforms are distinguished by their C-terminal sequences, which include a serine-rich region (polyS-domain) in JMJD6-2 that is not present in JMJD6-Ex5. Immunoprecipitation followed by LC-MS/MS for JMJD6-Ex5 shows that different sets of proteins interact with JMJD6-2 and JMJD6-Ex5 with only a few overlaps. In particular, we found TFIIF-associating CTD phosphatase (FCP1), proteins of the survival of motor neurons (SMN) complex, heterogeneous nuclear ribonucleoproteins (hnRNPs) and upstream binding factor (UBF) to interact with JMJD6-Ex5. Like JMJD6-2, both UBF and FCP1 comprise a polyS-domain. The polyS domain of JMJD6-2 might block the interaction with polyS-domains of other proteins. In contrast, JMJD6-2 interacts with many SR-like proteins with arginine/serine-rich (RS)-domains, including several splicing factors. In an HIV-based splicing reporter assay, co-expression of JMJD6-2 inhibited exon inclusion, whereas JMJD6-Ex5 did not have any effect. Furthermore, the silencing of *jmjd6* by siRNAs favored *jmjd6-Ex5* transcripts, suggesting that JMJD6 controls splicing of its own pre-mRNA. The distinct molecular properties of JMJD6-2 and JMJD6-Ex5 open a lead into the functional implications of the variations of their relative abundance in tumors.

## 1. Introduction

Jumonji domain-containing protein (JMJD6) is involved in many developmental processes including embryogenesis, angiogenesis and tumorigenesis [1,2,3]. Genetic knock-out experiments in mice showed severe developmental defects affecting the brain, heart, lung, kidney and colon of embryos on embryonic day E13.5 and E17.5 with perinatal death or death shortly after birth [1,4,5]. JMJD6 is a unique Fe(II)- and 2OG-dependent dioxygenase within the group of JmjC-domain-containing proteins. It has been reported to hydroxylate lysine residues and demethylate arginine residues in proteins and histones [6,7,8,9]. Recently, additional catalytic activities were proposed, namely a kinase and a protease function [10,11]. Among the substrates for JMJD6-hydroxylation activity is the U2AF 65 kDa subunit (U2AF65), a key regulator of splicing [6]. Interactions with other splicing factors like Luc7-like 2 (Luc7l2) have also been described, and it was suggested that JMJD6 is involved in regulating alternative splicing [6,12]. Accordingly, the knock-down of JMJD6 in HEK-293T cells resulted in a significant change of the outcome for 14% of all alternative splicing events (*n* = 3710), and 74% of those were co-regulated with U2AF65 [13]. 

In addition to the catalytic JmjC domain, JMJD6 has a conserved AT-Hook sequence and a serine-rich domain (polyS) in its C terminus. The C-terminal region is involved in mediating protein–protein interactions, RNA-binding, subnuclear targeting and oligomerization of JMJD6 [12,14,15]. Special functions have been attributed to the polyS domain. It mediates the shuttling of JMJD6 between the nucleolus and the nucleoplasm. Moreover, transmission electron microscopy (TEM) studies have revealed that the oligomeric structure of JMJD6 changed from rings to fibrils when the polyS domain was deleted [15]. 

Many cancer types show an upregulation of JMJD6, which is associated with increased proliferation and invasion, causing aggressive tumors and poor prognosis (review by Yang et al. (2020)) [16]. This has been attributed to JMJD6 targeting a number of proteins involved in the regulation of gene expression (BRD4, N-Myc) and the cell cycle (p53) [17,18,19]. However, JMJD6 isoform-specific patterns in cancer have not been analyzed yet.

**Figure 1:**
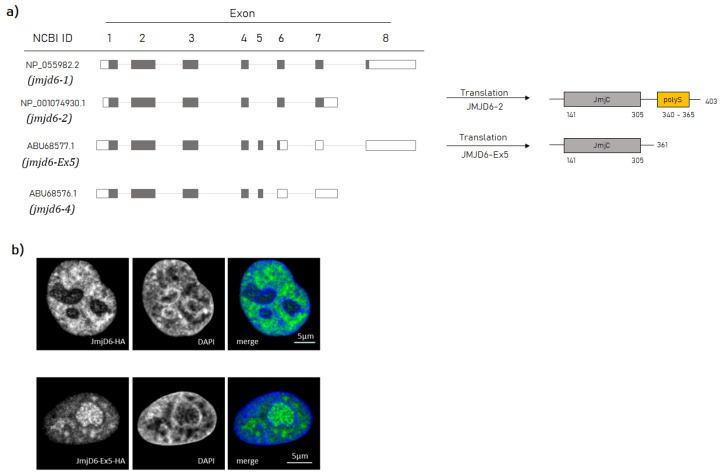
Structural and locational differences between the two isoforms JMJD6-2 and JMJD6-Ex5. (**a**) Comparison of the four annotated *jmjd6* transcripts from NCBI (left-hand site). While *jmjd6-1* only excludes exon 5, *jmjd6-2* excludes exon 5 and 8. In contrast, *jmjd6-Ex5* and *jmjd6-4* are including exon 5 in their transcripts. This leads to an earlier stop codon in the transcript. However, *jmjd6-4* uses an alternative donor site in exon 5 (coding exons are indicated in grey). Comparison of the protein sequence of JMJD6-2 and JMJD6-Ex5 (right-hand site). While both isoforms possess the catalytic JmjC domain (indicated in grey boxes), only JMJD6-2 carries a polyS domain in the C Terminus (indicated as orange box). (**b**) Confocal microscopic single sections of HeLa cells overexpressing JMJD6-2-HA (upper row) or JMJD6-Ex5-HA (lower row). Anti-Hemagglutinin (Anti-HA) tag antibody staining (green) and 4′,6-diamidino-2-phenylindole (DAPI) staining (blue) are shown together as a merged image as indicated. Scale bar: 5 µm.

The *jmjd6* gene consists of eight exons which can be alternatively spliced (Figure 1a). Four isoforms are listed in the NCBI database (NP_001074930.1, NP_055982.2, ABU68577.1, ABU68576.1). All protein isoforms possess the catalytic JmjC domain, but differ in their C-terminal region (Supplementary Figure S1). So far, most reported work has focused on isoform JMJD6-1 (NP_001074930.1) and JMJD6-2 (NP_055982.2). Both possess a conserved polyS domain in the C-terminus; however, JMJD6-1 translates for an additional 11 C-terminal amino acids, which is a result of exon 8 inclusion into the *jmjd6-1* transcript. Furthermore, *jmjd6-1* and *jmjd6-2* transcripts exclude exon 5. Exon 5 inclusion results in a frameshift in exon 6, exposing a premature stop codon. This gives rise to the protein isoform 3 (ABU68577.1) with an alternative C-terminal sequence lacking a polyS-domain (Figure 1a). A fourth transcript also includes exon 5 but uses an alternative splice donor site, which translates into isoform 4 (ABU68576.1). *TCGA SpliceSeq* data indicate that exon 8 inclusion (*jmjd6-1*) and exon 5 inclusion with an alternative donor site (*jmjd6-4*) are very rare events [20]. In contrast, *jmjd6-2* transcripts are the most abundant and *jmjd6-3* transcripts have been found in many human tissues and cell lines [15,21]. Sequencing cDNA from their 3′ ends and a search at the *PolyASite* database revealed that both transcripts use the same polyadenylation site in intron 7 [15,22]. This sparked our interest in its molecular function.

In the present study, we, therefore, compared JMJD6-3 (ABU68577.1) with JMJD6-2 (NP_055982.2). To stress the critical exon 5 inclusion in *jmjd6-3* transcripts we use the terms *jmjd6-Ex5* (transcript) or JMJD6-Ex5 (protein) for this isoform, but we keep the names *jmjd6-2* (transcript) and JMJD6-2 (protein) for isoform 2.

As previous studies had indicated, JMJD6-2 is localized in the nucleoplasm, whereas JMJD6-Ex5 localizes in the nucleolus and in nuclear speckles (Figure 1b) [15]. Results of our present work revealed that both isoforms are differentially regulated in cancer tissue, engaged in very distinct protein–protein interactions, and accordingly differed in their nuclear functions. Moreover, it appeared that JMJD6 feeds back on the splicing of its own pre-mRNA.

## 2. Results

### 2.1. Jmjd6-Ex5 Is Significantly Decreased in JMJD6 Relevant Cancer Types

**Figure 2:**
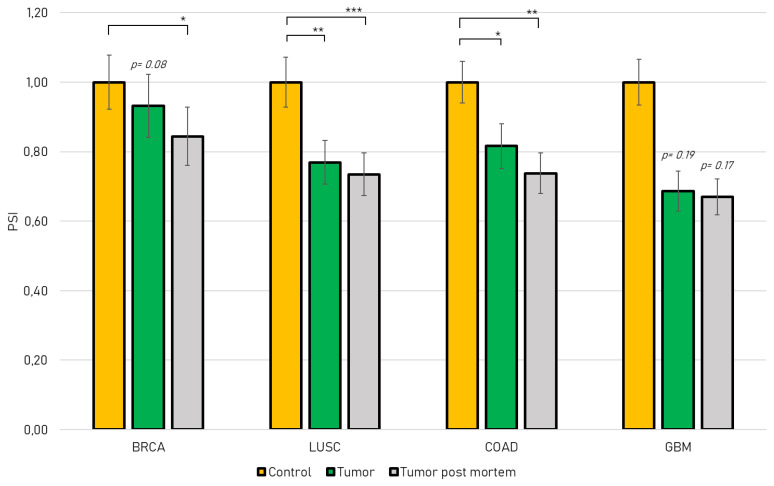
*TCGA SpliceSeq* Database search showing Percent Spliced In values (PSI) of *jmjd6* exon 5 (resulting in JMJD6-Ex5) in Breast Invasive Carcinoma (BRCA), Lung Squamous Cell Carcinoma (LUSC), Colon Adenocarcinoma (COAD) and Glioblastoma (GBM). Tumor samples from patients (green bars) and samples from *post-mortem* patients (grey bars) were normalized to a healthy control group (yellow bars). Mean values ± SEM are presented and a t-test was conducted (* *p* < 0.05, ** *p* < 0.01, *** *p* < 0.001).

JMJD6 expression is correlated with tumor progression, invasiveness and metastasis in several cancer types and is elevated in advanced tumors [16]. We used the *TCGA SpliceSeq* database to study the differential abundance of *jmjd6* exon 5 inclusion in four Jmjd6 relevant cancer types [20]: Breast Invasive Carcinoma (BRCA) [23], Lung Squamous Cell Carcinoma (LUSC) [24], Colon Adenocarcinoma (COAD) [19] and Glioblastoma (GBM) [25]. All four cancer types show a change in *jmjd6* alternative transcript expression with *jmjd6-Ex5* being less abundant. In detail, we show that exon 5 inclusion is significantly decreased in Lung Squamous Cell Carcinoma and Colon Adenocarcinoma compared to healthy tissue (Figure 2). Data from *post-mortem* tumors revealed a significant decrease of exon 5 inclusion in Breast Invasive Carcinoma, Lung Squamous Cell Carcinoma and Colon Adenocarcinoma. In addition, tissue from Glioblastoma indicated a decrease of exon 5 inclusion as well, although in this case with low significance (*p* > 0.19). This analysis suggests a differential role for JMJD6 alternatively spliced isoforms in tumor progression. We therefore investigated the molecular properties of the predicted protein isoforms JMJD6-2 and JMJD6-Ex5. 

### 2.2. Protein–Protein Interactions of JMJD6-2 and JMJD6-Ex-5 and the Role of the polyS-domain

**Figure 3:**
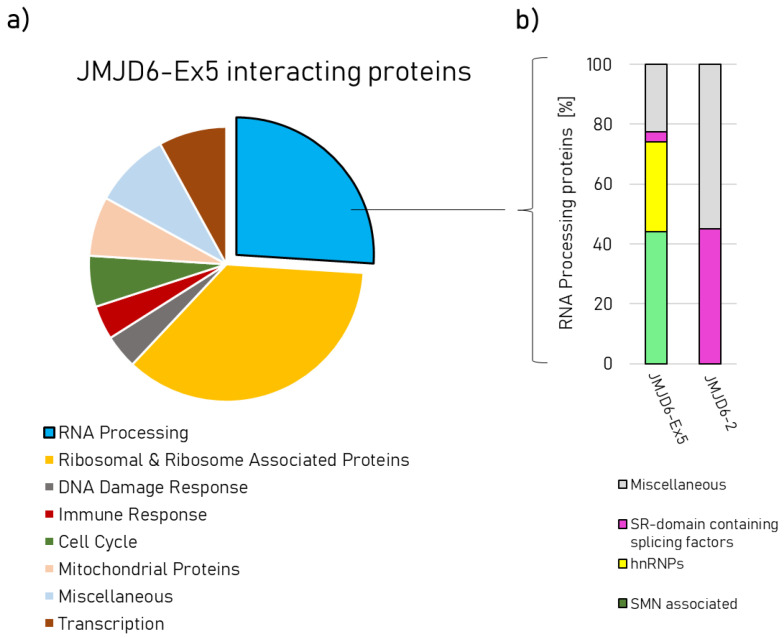
(**a**) Graphic representation of manually assigned functions for 101 proteins co-immunoprecipitated with HA-tagged JMJD6-Ex5 protein. (**b**) Specific assignments for the group of RNA-processing proteins co-immunoprecipitated by JMJD6-Ex5 (left-hand column) and JMJD6-2 (right-hand column, information from (Heim et al. (2014) [12]).

While JMJD6-2 is localized in the nucleoplasm, JMJD6-Ex5 is located in nuclear speckles and in the fibrillary centers of the nucleoli (Figure 1b) [15]. This suggests that these isoforms are present in different nuclear protein complexes. Whilst previous extensive protein interaction screens had revealed many protein–protein interactions for JMJD6-2 [12] we now sought to identify protein interactions for JMJD6-Ex5. To this end, we carried out an immunoprecipitation experiment (IP) in HeLa–cells using HA-tagged JMJD6-Ex5 as bait. The precipitate was analyzed by liquid chromatography mass spectrometry (LC-MS/MS). We found 101 significantly enriched proteins compared to control IP (see Supplementary dataset Table S1). Two major fractions constituted RNA processing proteins (26/101) and ribosome-associated proteins (36/101) (Figure 3a). Comparing the JMJD6-Ex5 IP-results with previously published JMJD6-2 interacting proteins revealed strong differences [12]. The JMJD6-2 interactome contains many serine/arginine (SR)-like proteins with arginine/serine-rich (RS) domains. Those were not present in the JMJD6-Ex5 immunoprecipitate. Instead, we found proteins of the survival of motor neurons (SMN) complex and heterogeneous nuclear ribonucleoproteins (hnRNPs) (Figure 3b). The most abundant protein in the JMJD6-Ex5 IP was TFIIF-associating CTD phosphatase (FCP1)—a polyS-domain-containing protein [26]. However, comparing our JMJD6-Ex5 IP results with previously published data for JMJD6-2 can only serve as an indicator of differential interactions. Therefore, we proceeded to do a direct comparison of Co-IPs for JMJD6-Ex5 and JMJD6-2 with selected proteins to prove the aforementioned differences in interaction. 

**Figure 4:**
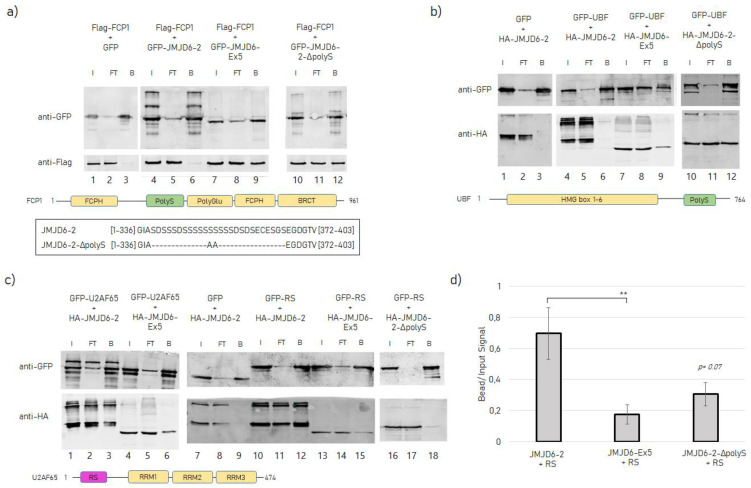
Co-immunoprecipitation experiments for interactions of JMJD6 variants with FCP1, UBF, U2AF65 and U2AF65- arginine/serine-rich (RS)-domain only, performed in HEK 293T cell lysates 48 h after transfection of respective plasmids; Input (I), flow-through (FT) and bound on beads (B) fractions are shown after SDS-PAGE and Western Blotting. Western Blots were stained with antibodies as indicated on the left-hand side of the Blots. (**a**) Flag-tagged FCP-1 and GFP-tagged JMJD6 variants; Bottom part shows a schematic presentation of the protein structure of FCP1 with its serine-rich region highlighted in green (PolyS). Underneath is the sequence of polyS-domain in JMJD6-2 in comparison with mutant JMJD6-2-ΔpolyS. (**b**) GFP-tagged UBF and HA-tagged JMJD6 variants; Bottom part shows a schematic presentation of the protein structure of UBF with its serine-rich region highlighted in green (PolyS). (**c**) GFP-tagged U2AF65 and HA-tagged JMJD6 variants plus GFP-tagged RS-domain of U2AF65 (U2AF65_20-70_, RS) and HA-tagged JMJD6 isoforms. Bottom part shows a schematic presentation of the protein structure of U2AF65 with its arginine- and serine-rich region highlighted in pink (RS). (**d**) Ratio between band intensity of input and bead signal from IPs (*n* = 7) with JMJD6-2, JMJD6-Ex5 and JMJD6-2-ΔpolyS and GFP-RS, analyzed with ImageJ. Mean values ± SEM are presented and a t-test was conducted (** *p* < 0.01).

In order to confirm the specific protein–protein interaction of JMJD6-Ex5 with FCP1 we performed co-IP assays in human embryonic kidney cells (HEK 293T). Flag-tagged FCP1 was co-expressed with green fluorescent protein (GFP) -tagged JMJD6-Ex5 or JMJD6-2, and anti-GFP-IP was carried out (Figure 4a). This revealed that Flag-FCP1 was co-precipitated with GFP-JMJD6-Ex5 but not with GFP-JMJD6-2 (Figure 4a, lane 4–9). However, when we deleted the polyS-domain from GFP-JMJD6-2, the interaction of the resulting JMJD6 mutant (JMJD6-2-ΔpolyS, find the sequence in Figure 4a, bottom part) with FCP1 did occur (Figure 4a, 10–12) suggesting that the polyS-domain of JMJD6-2 blocks binding of JMJD6-2 to FCP1. 

An inhibitory effect of the polyS-domain of JMJD6-2 on a protein–protein interaction was next shown for the RNA polymerase I transcription factor upstream binding factor (UBF). Previous studies had indicated co-localization of JMJD6-Ex5 in the fibrillary center of the nucleolus [15]. Like FCP1, UBF has a polyS-domain as well. We therefore co-expressed GFP-tagged UBF with HA-tagged JMJD6-Ex5, JMJD6-2 or JMJD6-2-ΔpolyS in HEK 293T-cells and performed anti-GFP-IP (Figure 4b). GFP-UBF co-precipitated JMJD6-Ex5 and JMJD6-2-ΔpolyS (Figure 4b, lane 7–12), but not JMJD6-2 (Figure 4b, lane 4–6), suggesting that the polyS-domain of JMJD6-2 blocked the interaction with UBF. In conclusion, the presence or absence of the polyS-domain in JMJD6 isoforms has a profound effect on their engagement in specific protein–protein interactions. 

We then set out to test whether the protein–protein interactions of JMJD6-2 with SR-like proteins were also isoform-specific. We co-expressed GFP-tagged U2AF65, an RS-domain-containing protein with HA-tagged JMJD6-2 or JMJD6-Ex5 in HEK 293T-cells and performed anti-GFP-IP (Figure 4c). This revealed that JMJD6-2 interacted more strongly with GFP-U2AF65 than JMJD6-Ex5 (Figure 4c, lane 1–6). Next, we used the sole RS-domain of U2AF65 (U2AF65_20-70_, RS) for this experiment [12]. Co-IP (Figure 4c, lane 10–18) and quantification of the Western Blot signals revealed that significantly more JMJD6-2 than JMJD6-Ex5 or JMJD6-2-ΔpolyS were co-precipitated by GFP-U2AF65_20-70_ (Figure 4d). These data suggest that the C terminus of JMJD6-2 including its polyS-domain increases the interaction of JMJD6-2 with SR-like proteins. 

Together these results indicate that the two JMJD6-isoforms, JMJD6-2 and JMJD6-Ex5 are engaged in different protein interactions in the nucleus. This is supported by their drastically different subnuclear distribution [15]. Moreover, the polyS-domain appears to be important for these differences. We next investigated whether this had functional implications by analyzing the effects of both isoforms on splice site selection.

### 2.3. JMJD6-Ex5 Is not Involved in Splice Site Selection of a Reporter Gene

**Figure 5:**
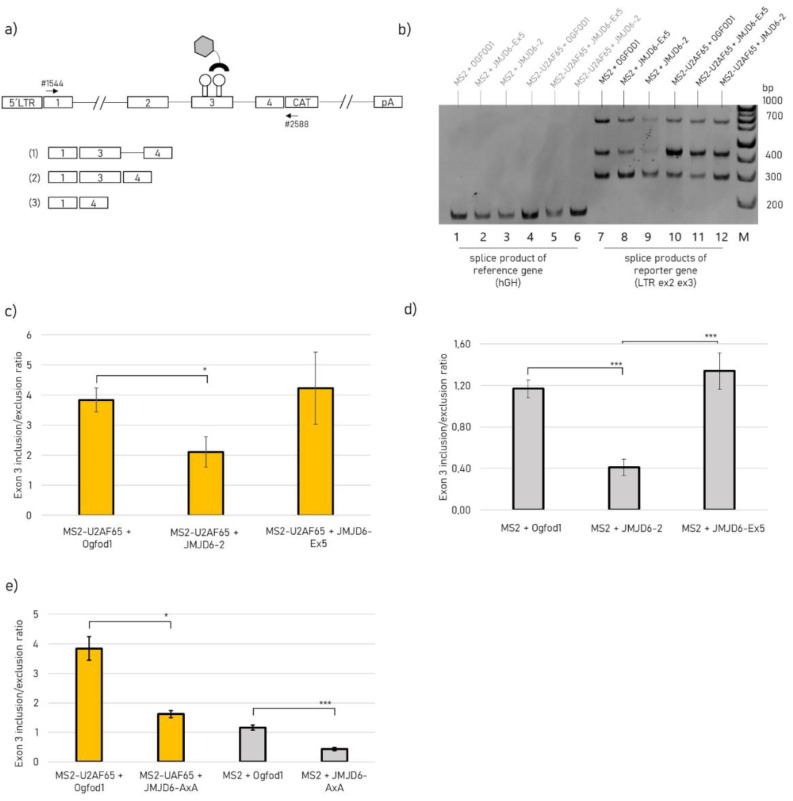
HIV-1-based splicing reporter with JMJD6-2, JMJD6-Ex5 and the catalytic inactive mutant JMJD6-AxA. (**a**) Schematic image of the LRT ex2 ex3 reporter gene construct with MS2 binding sites within exon 3 and the primers #1544 and #2588 marked with arrows. Image modified from Singh et al. (2010) [27]. The three possible splicing products that result from differential usage of the HIV-1 exon 3 splice sites SA2 and SD2 are indicated below and were separated by (**b**) a native PAGE after amplification of the splicing products using PCR. Of note, no inclusion of exon 2 into the reporter mRNAs can be detected due to weak intrinsic strength of HIV-1 exon 2 5′ss D2 and deletion of splice enhancing sequences upstream of exon 2 for intron shortening. Human Growth hormone (hGH) splice product is used as a reference. Sample loading was as followed: lane (1) MS2 + Ogfod1; (2) MS2 + JMJD6-Ex5; (3) MS2 + JMJD6-2; (4) MS2-U2AF65 + Ogfod1; (5) MS2-U2AF65 + JMJD6-Ex5; (6) MS2-U2AF65 + JMJD6-2; (**c**–**e**) ImageJ quantification of spliced products separated on native PAGE-gels after normalization with hGH mRNA bands. Ratio for exon 3 inclusion/exclusion was calculated for each condition. The Fe(II) and 2OG dependent oxygenase Ogfod1 (ID: Q8N543), which is not involved in splicing was overexpressed for control. Mean values ± SEM from 7 experiments are presented and a t-test was conducted (* *p* < 0.05, *** *p* < 0.001).

To investigate the effect of JMJD6 isoforms on splice site selection we used a HIV-1-based reporter gene with an MS2-binding site within exon 3 (Figure 5a). For this, we have weakened the strength of the exon 3 donor at the expense of exon 2 recognition. Although this leads to a weaker exon 3 recognition, it is at the same time associated with a greater possibility of regulating exon 3 recognition. The MS2 stem loops allow tethering of MS2 coat protein-tagged proteins to the test exon 3 and assess their effect on splice site usage [27]. Alternative splicing of the reporter pre-mRNA results in three different mRNA products that are formed by differential activation of the exon 3 flanking splice sites and can either include or skip test exon 3. The mRNA product of the co-transfected human growth hormone is used as a reference (Figure 5b, lane 1–6). We analyzed isoform JMJD6-2 and JMJD6-Ex5 regarding its involvement in splice site selection and included an active site mutant of JMJD6, which reflects an enzymatically inactive protein (JMJD6-AxA). In the absence of co-expression of the MS2 fusion protein, we could detect approximately similar levels of exon 3- inclusion and exclusion (Figure 5b, lane 7). Tethering U2AF65-MS2 to exon 3 substantially increased its inclusion four-fold (Figure 5c). However, JMJD6-2 overexpression significantly diminished U2AF65-MS2 induced exon 3 inclusion, while JMJD6-Ex5 did not change the splicing outcome. Likewise, in the absence of U2AF65-MS2 tethering a significant decrease in exon 3 inclusion was also seen, when only co-expressing JMJD6-2 but not JMJD6-Ex5 (Figure 5d). Although not expected *a priori*, this suggests that JMJD6-2, despite its C-terminal domain which can interact with SR- like proteins and their RS domains, can negatively influence exon recognition. 

Since U2AF65 is considered a substrate for JMJD6 hydroxylation activity, we asked if the catalytic activity of JMJD6-2 might have rendered U2AF65 splicing inactive. Therefore, we repeated the transfection experiment using a catalytic inactive mutant of JMJD6-2, called JMJD6-AxA in the following [28]. However, co-expression of JMJD6-AxA with the splicing reporter also led to a significant decrease in exon 3 inclusion, comparable to the JMJD6-2 wildtype levels independent of U2AF65-MS2 tethering (Figure 5e). In summary, these results indicate that JMJD6-2 inhibits exon 3 inclusion in this splicing assay independent of its catalytic activity and of U2AF65-MS2. For JMJD6-Ex5 we did not see any involvement in splice site regulation. 

### 2.4. JMJD6 Regulates Splicing of Its Own Gene to Promote Exon 5 Exclusion

**Figure 6:**
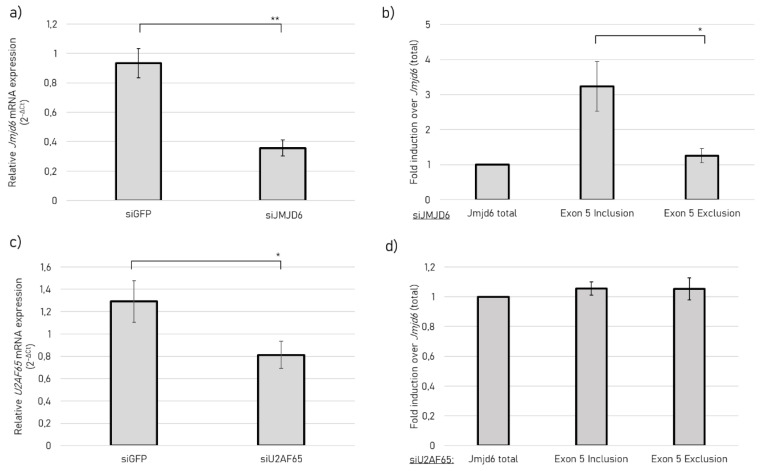
Exon 5 splicing at *Jmjd6* locus. (**a**) *Jmjd6* expression in HEK 293nT cells after transfection with siGFP (control) or siJmjd6 after RT-qPCR; *Cyclophilin A* was used as a reference gene for RT-qPCR to calculate relative *jmjd6* mRNA expression after transfection; (**b**) Fold induction of Exon 5 inclusion and exclusion transcripts over *jmjd6* after *jmjd6* siRNA transfection, total *jmjd6* was used as reference gene for RT-qPCR to calculate fold change expression of *jmjd6-2* (Exon 5 exclusion) and *jmjd6-Ex5* (Exon 5 inclusion). (**c**) *U2af65* expression in HEK 293nT cells after transfection with siGFP (control) or siU2AF65. *Cyclophilin A* was used as a reference gene for RT-qPCR to calculate relative *u2af65* mRNA expression after transfection. (**d**) Fold induction of Exon 5 inclusion and exclusion transcripts over *Jmjd6* after *U2af65* siRNA transfection, total *Jmjd6* was used as a reference gene for RT-qPCR to calculate fold change expression of *jmjd6-2* (Exon 5 exclusion) and *jmjd6-Ex5* (Exon 5 inclusion). Mean values ± SEM from three experiments are presented and a t-test was conducted (* *p* < 0.05, ** *p* < 0.01).

Given the impact of JMJD6-2 on the repression of exon inclusion, we queried whether JMJD6 might regulate exon 5 recognition of its own pre-mRNA. Therefore, we performed RNA interference (RNAi) experiments to silence JMJD6 expression, quantified endogenous *jmjd6-2* and *jmjd6-Ex5* transcripts and related this to total *jmjd6* mRNA in HEK-293nT cells. We used esiRNA, which contains multiple siRNAs, targeting *jmjd6* exon 2. This exon is present in all transcribed *jmjd6* isoforms and ensures a specific knock-down. Silencing *jmjd6* by 60% (Figure 6a) promoted exon 5 inclusion (Figure 6b), resulting in an increase of *jmjd6-Ex5* transcripts. Since JMJD6 shares some of its targets with U2AF65, we asked whether U2AF65 is also a regulator of *jmjd6* alternative splicing. Therefore, we silenced *U2af65*. However, silencing *U2af65* (Figure 6c) did not change the splicing outcome (Figure 6d). This suggests that JMJD6 autoregulates exon 5 recognition, which seems to be independent of U2AF65.

## 3. Discussion

The Fe(II) and 2OG dependent oxygenase JMJD6 is an important factor for embryonic development and it is strongly indicated in controlling tumor progression. The underlying molecular mechanisms are not always well understood but different lines of evidence suggest that JMJD6 controls several aspects of gene expression in a context-dependent manner [29]. There are four isoforms of JMJD6 resulting from alternative splicing of the *jmjd6* pre-mRNA. Here, we compared two of the resulting protein isoforms, JMJD6-2 and JMJD6-Ex5. The starting point for this study was an analysis of the occurrence of these two transcript isoforms in human tumor tissue. JMJD6-Ex5 was expected to be degraded by the NMD pathway, which selectively degrades mRNAs harboring premature termination codons (PTCs) [30]. Nevertheless, the detectability of JMJD6-Ex5 was confirmed in our previous work [15] and in *TCGA SpliceSeq* data [20]. Indeed, *TCGA SpliceSeq* data showed a significant but relatively small decrease in Jmjd6-Ex5 in *post mortem* tissue of BRCA, COAD and LUSC, which links JMJD6-2 to cancer progression. Thus, different functions of the two protein isoforms encoded by two alternatively spliced transcripts might be of importance. 

Furthermore, we show that the protein interactome for JMJD6-Ex5 is significantly different from several published JMJD6-interactomes [12]. In all of the latter, a large number of SR-like proteins had been found and our own work had previously indicated that JMJD6-2 interacted with the RS-domains of those proteins [12]. Moreover, lysine residues in peptides derived from such RS-domains are hydroxylated by JMJD6 in vitro [6]. We had therefore speculated that these RS-domains might be substrates for the lysine-5-hydroxylase activity of JMJD6 and that they interacted with the catalytic JmjC-domain. This domain is present in both isoforms. Surprisingly, in our IP-screen with JMJD6-Ex5, SR-like proteins were not detected. Moreover, in direct Co-IPs, we found that JMJD6-Ex5 interacted only weakly with RS-domains in comparison with JMJD6-2. Thus, we conclude, that JMJD6-2 must have an additional binding site for RS-domains in its C-terminal region, apart from the catalytic site within the JmjC-domain. This binding site is not present in the alternative C-terminal sequence of JMJD6-Ex5; hence, interaction is diminished. 

**Figure 7:**
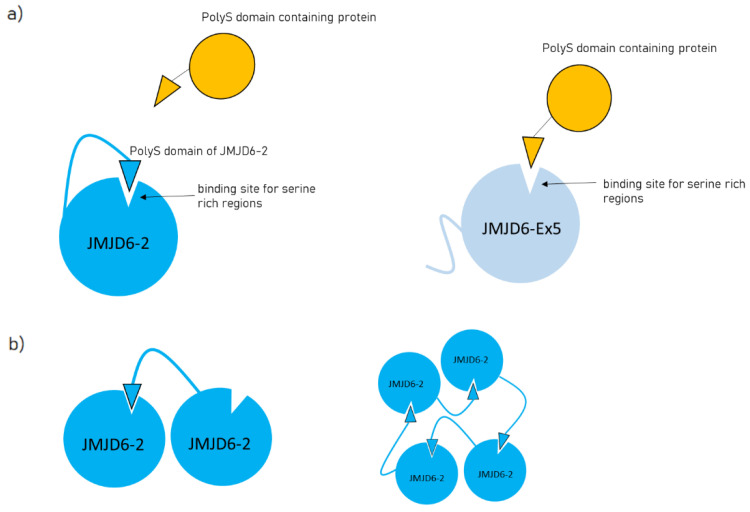
Proposed model of a polyS-domain binding site within JMJD6. (**a**) JMJD6-2 possesses a polyS domain in the C-terminal region and interacts with its own polyS binding site, thereby blocking interaction with other polyS domain-containing proteins. In contrast, JMJD6-Ex5 does not have its own polyS domain, allowing interaction with other polyS domain-containing proteins. (**b**) Interaction of JMJD6-2 to its own polyS binding site could be intermolecular and supporting oligomerization of JMJD6-2.

The major protein pulled down with JMJD6-Ex5 was FCP1 (TFIIF-associating CTD phosphatase). FCP1 dephosphorylates Ser2 and Ser5 of the C-terminal domain of RNA polymerase II and is thus involved in mRNA-transcription and in the DNA damage response [31,32,33]. We confirmed the interaction of FCP1 with JMJD6-Ex5 in an independent immunoprecipitation experiment. This experiment showed that JMJD6-2 did not interact with FCP1. We could attribute this failure to an inhibitory function of the polyS-domain of JMJD6-2. When we deleted this domain, JMJD6-2-ΔpolyS strongly interacted with FCP1. Interestingly, we obtained the same results with UBF. UBF has a C-terminal polyS-domain; it interacts only with JMJD6-2-ΔpolyS and JMJD6-Ex5 but not with JMJD6-2. From these data, we conclude that the polyS-domain of JMJD6-2 blocks the interaction with polyS-domains of other proteins. We speculate that a specific polyS-binding site is present in the JMJD6-molecule and this is covered by the C-terminal polyS-domain of JMJD6-2 (Figure 7a). Cryo-EM micrographs have shown that JMJD6-2 forms large distinct oligomers and the deletion of its polyS domain changed the oligomeric structure from rings to fibrils [15]. Therefore, binding of its own polyS-domain to a site within the JMJD6-molecule could be intermolecular, possibly promoting assembly of homo-oligomers (Figure 7b). When the JMJD6-polyS-domain is not present, oligomerization might be abolished and the polyS binding site is free to interact with polyS-domains of other molecules, e.g., UBF or FCP1. Nevertheless, to confirm this hypothesis further investigations are required. 

The JMJD6-Ex5 interactome also included hnRNP proteins, which can directly bind to the pre-mRNA and in a context-dependent manner either block specific splice sites or make them more accessible for the spliceosome [34]. Moreover, JMJD6-Ex5 pulled down several components of the SMN-complex, which is involved in assembling together the spliceosome [35]. Therefore, we tested whether JMJD6-Ex5 is involved in alternative splice regulation as it had been shown to be the case for JMJD6-2 in several studies [2,3,12,13]. We monitored the splicing of a HIV-1-based splicing reporter and found that JMJD6-2 significantly changed the splicing outcome by inhibiting exon inclusion. This was independent of tethering U2AF65 to the splice site, which by itself increased exon inclusion enormously, as had been demonstrated before [13]. In the presence or absence of U2AF65-MS2, JMJD6-2 decreased exon inclusion by half. The catalytic inactive mutant of JMJD6-2, JMJD6 AxA, did show similar results. This suggests that JMJD6-2 represses exon inclusion that does not involve its hydroxylase activity or its interaction with U2AF65-MS2. It is in accordance with the aforementioned study by Yi et al. (2017) showing that 26% of JMJD6 regulated alternative splicing events were not co-regulated with U2AF65 and 36% of JMJD6 induced exon skipping events were independent of its enzymatic activity [13]. Moreover, the isoform JMJD6-Ex5, which does not bind to SR-like proteins, does not have an effect on the splicing of this reporter at all. This confirms our conclusion that JMJD6-2 and JMJD6-Ex5 have distinct functions in the nucleus, which is in agreement with their separate subnuclear localisation and their almost exclusive protein interactomes. Therefore, the relative abundance of these JMJD6 isoforms is important for *jmjd6*-gene functions in the nucleus.

Thus, it was especially interesting to see whether JMJD6 was involved in determining the amounts of its own isoforms that accumulate in different nuclear compartments. Knocking down *jmjd6* led to a significant increase of exon 5 inclusion, resulting in increased *jmjd6-Ex5*-trancripts. This indicates that JMJD6 regulates itself via a feedback mechanism and shows another splicing event, in which JMJD6 promotes exon skipping. In addition, *jmjd6* exon 5 splicing regulation did not seem to be dependent on U2AF65. HEXplorer [36] analysis of *jmjd6* exon 5 indicates a binding site for hnRNPs, suggesting a regulatory mechanism of exon 5 inclusion/exclusion by hnRNP family members. In this context, our finding of several hnRNP proteins as potential interactors for JMJD6-Ex5 could be a lead to investigate the mechanism for the observed autoregulatory effect of JMJD6 on exon 5 splicing. 

In conclusion, this work indicates that JMJD6-2 and JMJD6-Ex5 have drastically different functions despite the fact that they both contain the enzymatic JmjC-domain. The differing C-terminal regions of the JMJD6-2 isoform seem to be instrumental in mediating protein–protein interactions. While JMJD6-2, as previously reported by several authors, interacts with SR-like proteins and is involved in splice regulation (amongst other functions), JMJD6-Ex5 interacts with FCP1 and UBF, and is not involved in splice regulation of the HIV-1-based reporter gene. 

Future research should reveal the relevance of JMJD6-Ex5 interactions with FCP1, the SMN-complex and hnRNPs. Moreover, it would be interesting to see what signals regulate the production of different JMJD6-isoforms. 

## 4. Materials and Methods

### 4.1. Cell Lines and Culture Conditions

HeLa cells, human embryonic kidney (HEK) 293T cells and HEK 293nT (ATCC Number: CRL-1573) cells were cultured in Dulbecco’s modified Eagle’s medium (DMEM) supplemented with 10% fetal bovine serum (FBS, Biochrom, Berlin, Germany), 100 units/mL penicillin and 100 µg/mL streptomycin (Gibco, Carlsbad, CA, USA) at 37 °C, 5% CO_2_.

### 4.2. Co-Immunoprecipitation Experiments

HEK293T cells were transfected at a confluency of 60% with C-terminal HA-tagged JMJD6-2, JMJD6-Ex5 or JMJD6-2-ΔpolyS and GFP-tagged U2AF65, U2AF65_20-70_ or UBF (26672, Addgene, Watertown, MA, USA) using Lipofectamine 2000 (11668030, Thermo Fisher, Waltham, MA, USA). The same experiment was conducted with HEK293T cells transfected with GFP-tagged JMJD6-2, JMJD6-Ex5 or JMJD6-2-ΔpolyS and Flag-tagged FCP1. Beads with anti-GFP (ABIN509397, ChromoTek, Planegg-Martinsried, Germany) were used for immunoprecipitation as described in Webby et al. (2009) [6]. Western blots were stained with mouse anti-GFP antibody (11814460001, Roche, Basel, Switzerland), rabbit anti-HA antibody (H6908, Sigma Aldrich, St. Louis, MO, USA) and mouse anti-Flag antibody (F1804, Sigma Aldrich, St. Louis, MO, USA). 

### 4.3. Interactome Analysis

For interactome analysis, HeLa cells were transfected with HA-tagged JMJD6-Ex5 using PEI (1 mg/mL, pH 7.4). After 48 h cells were lysed and incubated with DynabeadsTM Protein G (10003D, Thermo Fisher, Waltham, MA, USA) previously coated with anti-HA antibody (H6908, Sigma Aldrich, St. Louis, MO, USA). After 24 h incubation, beads were washed and proceeded further for mass spectrometry. This revealed 101 co-immunoprecipitated proteins detected by LC-MS/MS. The co-immunoprecipitated proteins were grouped by their main function found on uniprot (https://www.uniprot.org/, asscess date: 15.02.2020) for schematic presentation. 

### 4.4. Proteomic Sample Preparation

IP samples with Laemmli buffer were heated for 10 min at 95 °C and centrifuged for 5 min at 14,000× *g*. The supernatants containing eluted proteins were digested using a modified FASP procedure [37]. After reduction and alkylation using DTT and IAA, the proteins were centrifuged on a 30 kDa cutoff filter device (Pall, Port Washington, NY, USA), washed thrice with UA buffer (8 M urea in 0.1 M Tris/HCl pH 8.5) and twice with 50 mM ammoniumbicarbonate. The proteins were digested for 2 h at room temperature using 0.5 µg Lys-C (Wako Chemicals, Neuss, Germany) and for 16 h at 37 °C using 1 µg trypsin (Promega, Mannheim, Germany). After centrifugation (10 min at 14,000× *g*) the eluted peptides were acidified with 0.5% TFA and stored at −20 °C.

### 4.5. LC-MS/MS Measurement and Analysis

LC-MS/MS analysis was performed on a LTQ Orbitrap XL mass spectrometer (Thermo Scientific, Waltheim, MA, USA) online coupled to an Ultimate 3000 nano-RSLC (Thermo Scientific, Waltheim, MA, USA) as described previously [38]. Tryptic peptides were automatically loaded on a C18 trap column (300 µm inner diameter (ID) × 5 mm, Acclaim PepMap100 C18, 5 µm, 100 Å, LC Packings) prior to C18 reversed phase chromatography on the analytical column (Acclaim PepMap C18, 50 µm ID × 250 mm, 2 µm, 100 Å, LC Packings) at 300 nL/min flow rate in a 140 min acetonitrile gradient from 4 to 30% in 0.1% formic acid. Profile precursor spectra from 300 to 1500 m/z were recorded in the orbitrap with a maximum injection time of 500 ms. TOP10 fragment spectra of charges 2 to 7 were recorded in the linear ion trap with a maximum injection time of 100 ms, an isolation window of 2.0 m/z, a normalized collision energy of 35 and a dynamic exclusion of 60 s.

Generated raw files were analyzed using Progenesis QI for proteomics (version 2.0, Nonlinear Dynamics, part of Waters) as described previously [38,39]. Features of charges 2–7 were used and all MSMS spectra were exported as mgf file. Peptide searches were performed using Mascot search engine (version 2.5.1) against the Ensembl Human protein database (100158 sequences, 37824871 residues). Search settings were: 10 ppm precursor tolerance, 0.6 Da fragment tolerance, one missed cleavage allowed. Carbamidomethyl on cysteine was set as fixed modification, deamidation of glutamine and asparagine allowed as variable modification, as well as oxidation of methionine. Applying the percolator algorithm [40] with a cut-off of 13 and *p* < 0.05 resulted in a peptide false discovery rate (FDR) of 1.54%. Search results were reimported in the Progenesis QI software. Proteins were quantified by summing up the abundances of all unique peptides per protein. Resulting protein abundances were used for calculation of fold-changes and statistics values.

### 4.6. HIV-1-Based Splicing Reporter Assay

Plasmids and primers were previously described in Singh et al. (2010) and Hildebrand et al. (2017) [27,41]. HeLa cells at 60–70% confluency were used for transfection with Lipofectamine^®^ 2000 (Invitrogen, Waltham, MA, USA) according to the manufacturer’s instructions. For each condition, cells were seeded in 6-well culture plates and co-transfected with the reporter gene construct LTR ex2 ex3 and pXGH5. The latter of which expresses constitutively spliced human growth hormone (hGH) mRNA to monitor transfection efficiency and viability of the cellular splicing apparatus. Control experiments included overexpression of Ogfod 1 (ID: Q8N543) and MS2. 30 h after transfection cells were collected and total RNA was isolated using RNeasy kit (74104, Qiagen, Venlo, The Netherlands). For reverse transcription 1.5 µg of total RNA was subjected to DNA digestion with 10 U of recombinant RNAse-free DNAse I (Roche, Basel, Switzerland) in total volume of 20 µL at 37 °C for 20 min following the inactivation of DNAse at 70 °C for 15 min. 9 µL of treated RNA was then mixed with 1 µL of Oligo-d(T)12–18 primer (Invitrogen, Waltham, MA, USA), 1 µL of 10 mM each dNTP mix (NEB, Ipswish, MA, USA) and 2 µL of RNAse-free water and incubated at 65 °C for 5 min. The samples were then cooled on ice for 1 min. 4 µL of 5xFirst Strand buffer, 1 µL 0.1M DTT, 1 µL (40 U) RNAsin (Promega, Madison, WI, USA ) and 10 µL (200 U) of SuperScript™ III reverse transcriptase (Thermo Fisher, Waltheim, MA, USA) was then added to the samples. Reverse transcription was performed at 50 °C for 1 h and 70 °C for 15 min. 5 µL of prepared cDNA were used as a template for specific PCR reactions. Amplification was performed in the total volume of 50 µL using 5 µL of 10× PCR buffer (Qiagen, Venlo, The Netherlands), 1 µL 10 mM each dNTP mix (NEB, Ipswish, MA, USA), 1 µL of each primer (10 pmol/µL) and 0.25 µL (1.25U) Taq DNA polymerase (Qiagen, Venlo, The Netherlands). PCR samples were mixed with loading buffer and 10 µL of samples were then loaded on 8% Native PAGE gels. The gels were run in 1x TBE buffer at 150 V for 1 h 10 min. The gels were stained after the run with HDgreen (Intas, Ahmedabad, India) for 10 min. PCR amplicons were visualized using Gel iX20 Imager (Intas, Ahmedabad, India) and quantified using ImageJ software. 

### 4.7. RNA Interference

A pool of four esiRNA oligomers per gene against JMJD6 and U2AF65 were purchased from Sigma (Sigma Aldrich, St. Louis, MO, USA). HEK293nT cells were grown to 20–30% confluence and transfected with esiRNA using TransIT-X2 transfection reagent following the manufacturer’s instructions (Mirus, Madison, WI, USA). After 24 h of incubation, cell culture media was refreshed and then incubated for an additional 48–72 h. Knockdown efficiencies were verified by qPCR. 

### 4.8. qRT-PCR from esiRNA Experiments

RNA was isolated from cells using the GENEzol TriRNA Pure Kit (GeneAid). cDNA synthesis was carried out with the Quanta cDNA Reverse Transcription Kit (QuantaBio). Then, qPCR was performed with the iTaq Supermix (BioRad, Hercules, CA, USA) on the Biorad iCycler. The comparative Ct method was employed to quantify transcripts, and delta Ct was measured in triplicate. 

Sequences of used primers were the following: Cyclophilin A (Fw: GTCAACCCCACCGTGTTCTT Rv: CTGCTGTCTTTGGGACCTTGT), U2AF65 (Fw: 5′ACCCAGGCTATGGCCTTTG, Rv: 5′GAAGCGGCTGGTAGTCGTG), Jmjd6 total (Fw: 5′GGGTGCGTTAGTGTCAGGAA, Rv: 5′CCTTTCCACGTTATCCGCCA), Jmjd6 Exon 5 Inclusion (Fw: 5′ACCTGGAGGGACCAGCTC, Rv: 5′TCTGAGTCGGAGTCTGACGA), Jmjd6 Exon 5 Exclusion (Fw: 5′CAAGGAAATGGTATAGGATTTTGAA, Rv: 5′TTTGCTGACACAGTCGTCCT). 

### 4.9. TCGA SpliceSeq Database

Data of JMJD6 isoforms distribution in cancer was downloaded from the TCGA SpliceSeq website (https://bioinformatics.mdanderson.org/public-software/spliceseq/, access date: 20.02.2020). TCGA SpliceSeq is an online free access resource for alternative splicing alterations in cancer, that provides data regarding alternatively spliced isoforms distribution—Percent Spliced In (PSI) values, as well as clinical TCGA data. Patients and Control PSI values were downloaded for JMJD6 alternative splicing events in specific cancer types: BRCA, COAD, LUSC, GBM. The data were grouped and analyzed regarding the supplied clinical data. Average PSI values were calculated and normalized to the control group.

## Supplementary Materials

Figure S1 and Table S1. Supplementary materials can be found at https://www.mdpi.com/1422-0067/21/18/6618/s1. Figure S1: Sequence Alignment of the four annotated JMJD6 isoforms from NCBI. Highlighted in green is the JmjC domain, which is present in all four isoforms and highlighted in yellow is a polyS domain only present in JMJD6-1 and JMJD6-2. Table S1: List of proteins identified via LC-MS/MS, which were significantly enriched after immunoprecipitation with HA-tagged JMJD6-Ex5 from HEK293T cells.
