# Supplementary figures and images for: JMJD6 Regulates Splicing of Its Own Gene Resulting in Alternatively Spliced Isoforms with Different Nuclear Targets
Source: Int J Mol Sci. 2020 Sep 10;21(18):6618. doi: 10.3390/ijms21186618 (PMC7555845; doi:10.3390/ijms21186618)

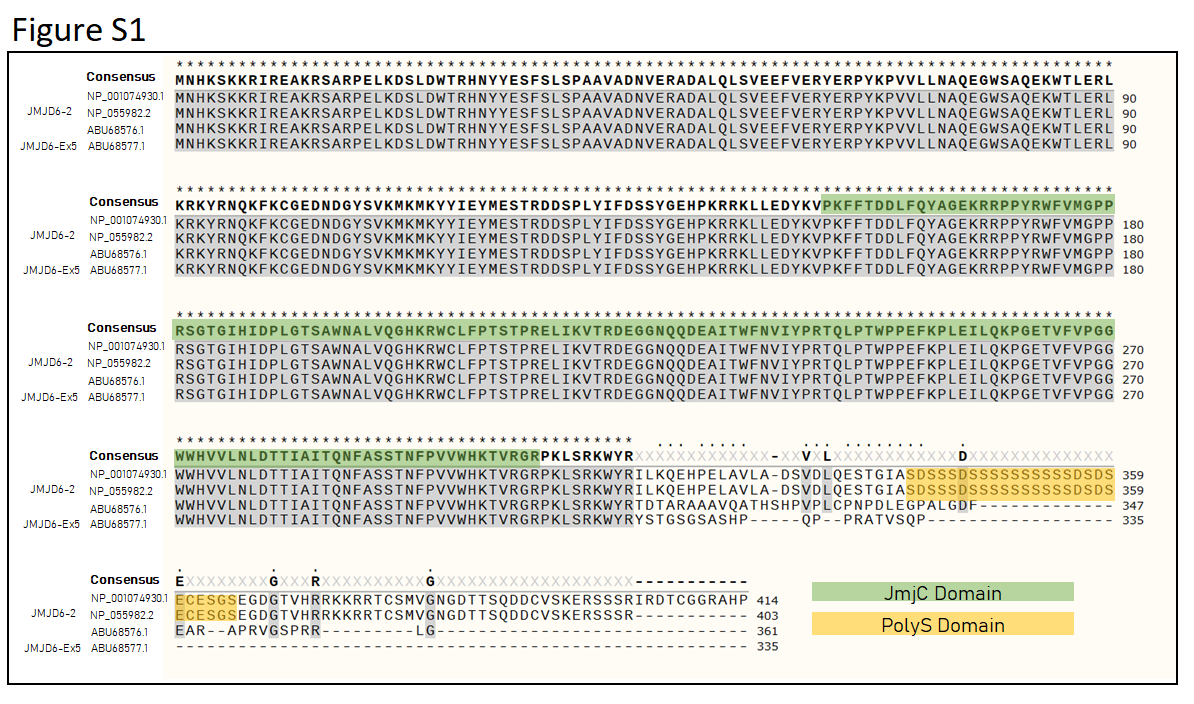

Supplement: Supplementary file 1 [file ijms-21-06618-s001.zip › Supplements/S1.tif]
